# Eintrübung von Intraokularlinsen: Erkenntnisse aus dem Labor und der Klinik

**DOI:** 10.1007/s00347-020-01259-3

**Published:** 2020-11-13

**Authors:** Ramin Khoramnia, Timur M. Yildirim, Grzegorz Łabuz, Christian S. Mayer, Gerd U. Auffarth

**Affiliations:** The David J Apple Center for Vision Research, Universitäts-Augenklinik Heidelberg, Im Neuenheimer Feld 400, 69120 Heidelberg, Deutschland

**Keywords:** IOL-Kalzifikation, Hydrophobes Acrylat, Hydrophiles Acrylat, Polymer, Glistenings, IOL calcification, Hydrophobic acrylate, Hydrophilic acrylate, Polymer, Glistenings

## Abstract

**Hintergrund:**

Das Spektrum der Komplikationen beim Einsatz von Intraokularlinsen (IOL) ist heute ein anderes als zur Zeit ihrer Einführung. Trübungen im IOL-Material werden vermehrt als Explantationsgrund in der Literatur beschrieben.

**Ziel der Arbeit:**

Diese Arbeit soll einen Überblick über die verschiedenen Formen von IOL-Materialeintrübungen geben.

**Material und Methoden:**

Die heute relevanten Formen von IOL-Materialeintrübungen und deren Auswirkungen auf die optische Abbildungsqualität wurden zusammengestellt.

**Ergebnisse und Diskussion:**

Als Grund für eine Eintrübung steht bei hydrophilen IOL die Kalzifikation im Vordergrund, während bei hydrophoben IOL v. a. über die Entstehung sog. „Glistenings“ berichtet wird. Die meisten Materialeintrübungen beeinflussen verschiedene optische Parameter und führen zu einem erhöhten intraokularen Streulicht. Im Falle einer störenden Materialeintrübung besteht die einzige Therapieoption in einem Austausch der eingetrübten IOL.

## Abstract (en)

**Background:**

Since their introduction the spectrum of complications of intraocular lenses (IOLs) has changed. Clouding of the IOL material is increasingly described in the literature as a reason for explantation.

**Objective:**

This study aimed to give an overview of the various pathologies of IOL material.

**Material and methods:**

The currently relevant types of IOL material opacities are summarized as well as their effects on the optical quality.

**Results and conclusion:**

In current IOL models calcification can occur in lenses made of hydrophilic acrylate, while in IOLs made of hydrophobic acrylate “glistenings” can develop. These different material opacifications have varying impacts on optical quality parameters and in general lead to an increased intraocular light scattering. In the case of intolerable IOL material opacification, the only treatment option is its removal and the implantation of a new IOL.

Durch Weiterentwicklungen in der Kataraktchirurgie haben sich das Komplikationsspektrum und die Explantationsgründe beim Einsatz von Intraokularlinsen (IOL) geändert. Berichte über Materialeintrübungen haben in der Literatur zugenommen. Bei hydrophilen IOL steht dabei die Kalzifikation im Vordergrund, bei hydrophoben IOL wird die Entstehung sog. „Glistenings“ beschrieben. Die verschiedenen Materialeintrübungen nehmen in unterschiedlichem Ausmaß Einfluss auf die optische Qualität. Trotz aller Fortschritte im Bereich der IOL-Materialien ist im Falle aller störenden Materialeintrübungen weiterhin ein Linsenaustausch die einzige mögliche Therapieoption.

## Verschiebung der Gründe für die Explantation von Intraokularlinsen

Die Kataraktoperation mit Implantation einer Kunstlinse ist eine der am häufigsten durchgeführten chirurgischen Eingriffe weltweit. Fortschritte in der Operationstechnik und die daraus resultierenden Verbesserungen der postoperativen Ergebnisse haben dazu geführt, dass die Operation zunehmend früher durchgeführt wird. Darüber hinaus nimmt aufgrund der höheren Lebenserwartung auch die voraussichtliche Dauer, die eine Intraokularlinse (IOL) im Auge verbleibt, zu. Die IOL-Implantation wird außerdem routinemäßig bei Patienten im Rahmen eines refraktiven Linsenaustausches durchgeführt und ist auch im Bereich der Behandlung der kindlichen Katarakt verbreitet [36]. In einigen dieser Fälle könnte die erwartete IOL-Verweildauer im Auge mehrere Jahrzehnte erreichen. Daher sollte das Material der Linse möglichst resistent gegen Veränderungen sein.

Die Gründe für die Notwendigkeit einer Explantation von Kunstlinsen haben sich seit ihrer Einführung geändert. In den 1980er- und 1990er-Jahren waren Entzündungsreaktionen, falsche IOL-Stärken und IOL-Dislokationen Hauptursachen für einen IOL-Austausch [4]. Obwohl diese Ursachen nach wie vor vorkommen und v. a. die IOL-Dislokationen noch für einen großen Anteil aller Explantationen verantwortlich sind, treten diese Gründe anteilmäßig zunehmend in den Hintergrund, da Eintrübungen von Kunstlinsen weltweit zugenommen haben. In einer kürzlich veröffentlichten Arbeit über 200 explantierte Kunstlinsen war in 76,5 % der Fälle die IOL-Trübung die Ursache für die Explantation und nur in 13,5 % die Dislokation [25]. Über Entzündungsreaktionen und eine falsche IOL-Stärke als Explantationsgrund wird heute kaum noch berichtet. Je nach Linsenmaterial finden sich typische Ursachen für eine Materialeintrübung. In Linsen aus rigidem Polymethylmethacrylat (PMMA) kann eine photochemische Materialdegradation auftreten und sich als schneeflockenartige Läsionen („snowflake degeneration“) präsentieren [3]. Nach Änderungen im Herstellungsprozess dieser Linsen wurde das Auftreten dieser Materialpathologie im Laufe der Zeit aber nur noch sehr selten beschrieben [30]. In IOL aus Silikon kann unter bestimmten Umständen eine Kalzifikation der Linsenrückfläche auftreten. Diese tritt jedoch ebenfalls sehr selten auf und steht meist im Zusammenhang mit einer asteroiden Hyalose [9]. Der weit überwiegende Anteil der heute eingesetzten IOL-Modelle besteht nicht mehr aus diesen Materialien, sondern aus einem faltbaren hydrophilen oder hydrophoben Acrylat. Dementsprechend werden heute eher andere Komplikationen beobachtet, welche im Zusammenhang mit diesen Materialien stehen. Während bei hydrophilen IOL v. a. eine Kalzifikation des Materials zur Eintrübung der Linse führen kann, überwiegt bei manchen Linsen aus hydrophobem Material das Auftreten von Glistenings [16].

## Kalzifikation in IOL aus hydrophilem Acrylat

Bei hydrophilen Acrylatkunstlinsen besteht die Gefahr, dass diese unter bestimmten Umständen kalzifizieren. Bei dieser Form der Trübung unterscheidet man klassisch zwischen einer primären und sekundären Form der Kalzifikation sowie einer Pseudokalzifikation [24].

### Primäre Kalzifikation

**Figure 1:**
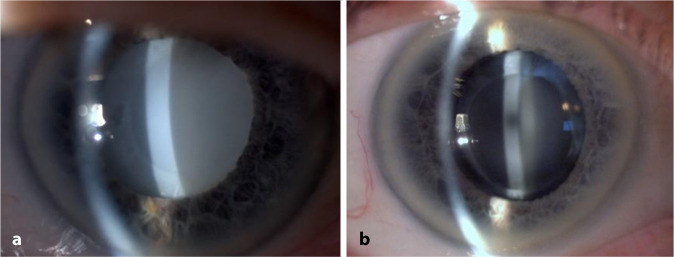

**Figure 2:**
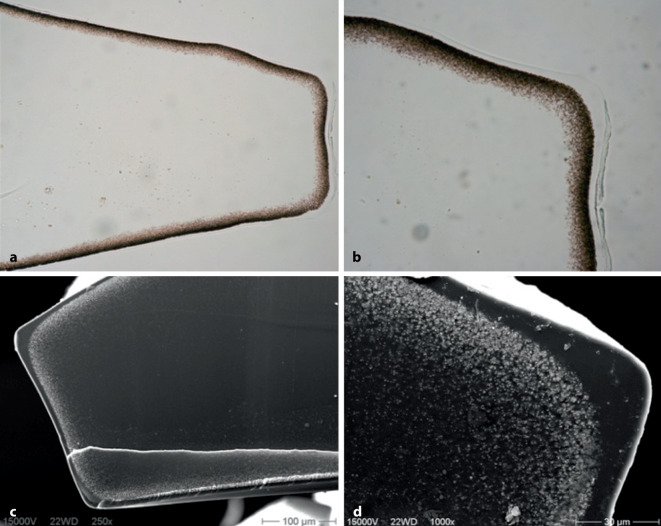

Die primäre Form der IOL-Kalzifikation bezieht sich auf Fälle, bei denen die Ursache für die Trübung in der Linse selbst liegt. So kann z. B. eine fehlerhafte Polymerfabrikation oder auch die Verpackung der IOL zu Eintrübungen führen [17, 31, 39]. Diese Art der Kalzifikation entsteht üblicherweise in Augen, bei denen keine Begleiterkrankungen bestehen. Vom Problem der primären Kalzifikation sind oftmals ganze Chargen betroffen. In der Literatur gibt es immer wieder Berichte über primäre Kalzifikationen in verschiedenen IOL-Modellen [10, 17, 25, 31, 32]. So beschrieben Izak et al. diese Pathologie beispielsweise in 25 IOL vom Typ Hydroview (Bausch & Lomb, Rochester, USA) und 54 IOL vom Typ SC60B-OUV (Medical Developmental Research, Clearwater, USA) [15]. Während bei diesen Linsen die Optik eine komplette Eintrübung zeigte, blieben die Haptiken klar. In Modellen des Typs Aqua-Sense (Ophthalmic Innovations International, Ontario, USA) zeigte sich hingegen eine komplette Kalzifikation der gesamten Linse inklusive der Haptiken (Abb. 1) [17]. Tehrani et al. berichteten 2004 von 6 eingetrübten hydrophilen IOL des Typs MemoryLens (Ciba Vision, Duluth, USA). Die mikroskopische Analyse ergab multiple, feine, körnige Ablagerungen unterschiedlicher Größe auf der Oberfläche der Linsenoptik [32]. In einer weiteren Laboruntersuchung wurden 6 Fälle explantierter primär kalzifizierter dreistückiger Euromaxx IOL (Argonoptics, Haltern am See, Deutschland) analysiert. Bereits in der Spaltlampenuntersuchung imponiert eine meist homogene Eintrübung der gesamten Linsenoptik, welche jedoch teilweise auch lokale Aussparungen aufweisen kann, so wie in der beschriebenen Studie meist am Ansatz der Haptiken. Wenn die Haptiken aus einem anderen Material als die Optik bestehen, muss also auch bei der primären Kalzifikation nicht die gesamte Linse von der Pathologie betroffen sein [31]. In den letzten Jahren wurde v. a. über die primäre Kalzifikation hydrophiler IOL-Modelle des Typs Lentis (Oculentis, Berlin, Deutschland) berichtet [25]. Gurabardhi et al. präsentierten 2018 klinische und lichtmikroskopische Ergebnisse von 71 Fällen, welche aufgrund einer primären Kalzifikation explantiert wurden. Die Serie betraf verschiedene Modelle aus den Jahren 2009 bis 2012 einschließlich der Modelle LS-502‑1, LS-402-1Y, LS-312-1Y, LS-313-1Y, L‑402 und L‑312 [14]. Aufgrund der Beliebtheit der Linsenmodelle nahm diese aktuelle Serie recht große Ausmaße an. So zeigte beispielsweise eine kürzlich veröffentlichte Arbeit von Scherer und Kollegen, dass es in 223 Augen, welche mit einer entsprechenden IOL versorgt wurden, in 67 Fällen, also in 30,0 % der Fälle, zu einer Eintrübung kam. Die Autoren konnten bis auf ein minimal erhöhtes Risiko bei älteren Patienten (OR=1,05) keine anderen begünstigenden patientenabhängigen Faktoren feststellen, die das Risiko einer Kalzifikation erhöhen [28]. Somit scheint v. a. die Produktionsweise der IOL ausschlaggebend für das Auftreten der Pathologie zu sein. Diese Problematik betraf auch die segmental refraktiven multifokalen Linsenmodelle derselben Firma, wie eine kürzlich veröffentlichte Arbeit zeigte [39]. Hier wurde über eine primäre Kalzifikation bei 8 Linsen der Modelle LS-313/2 MF30/15 berichtet [39]. Valide absolute Zahlen zur Inzidenz der Pathologie liegen uns nicht vor, da das alleinige Auftreten einer IOL-Kalzifikation nicht gemeldet wird und der Fall üblicherweise erst nach erfolgter Explantation registriert wird. Es ist deshalb anzunehmen, dass zusätzlich zu den bekannten Fällen eine unbekannte Dunkelziffer existiert. Dies liegt auch darin begründet, dass es bei vielen Patienten trotz Sehbeeinträchtigung nicht zu einem IOL-Austausch kommt, da z. B. entweder die Operation als zu risikoreich eingeschätzt oder vom Patienten nicht gewünscht wird. Makroskopisch zeigt sich bei der primären Kalzifikation in der Regel eine Eintrübung der Optik und ebenso der Haptiken, sofern diese aus demselben Material wie die Optik gefertigt sind. Licht- und rasterelektronenmikroskopisch können im Bereich der Eintrübung zahlreiche feine, granuläre Ablagerungen aus Kalziumphosphat nachgewiesen werden, die sowohl an der Vorder- als auch Rückfläche in einer wenige µm unterhalb der Oberfläche parallelen Linie angeordnet sind (Abb. 2).

### Sekundäre Kalzifikation

**Figure 3:**
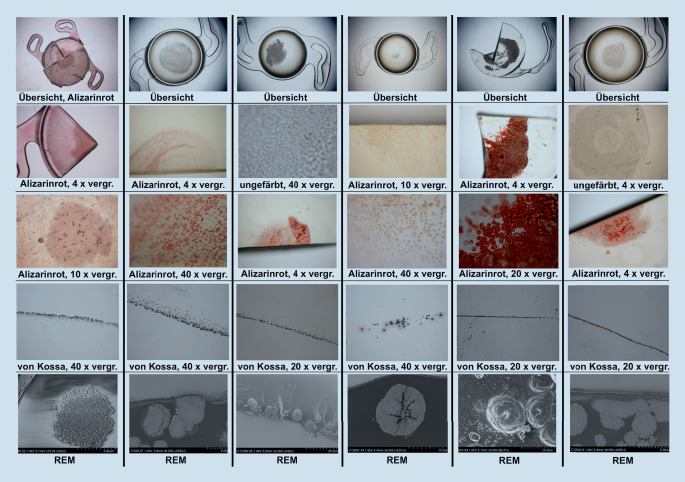

**Figure 4:**
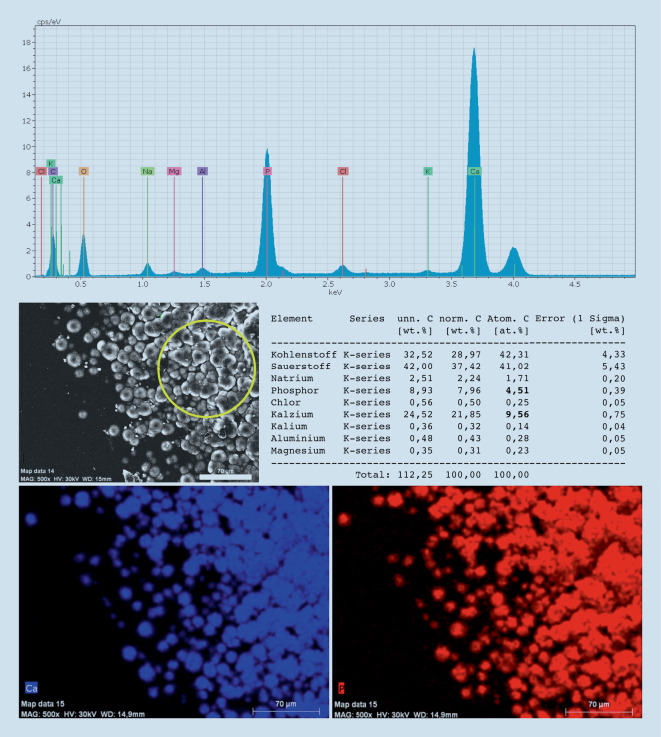

Die sekundäre Form der IOL-Kalzifikation wird am ehesten durch spezifische patientenseitig bestehende okuläre oder systemische Vorerkrankungen oder auch durch andere Operationen ausgelöst. Auch bei dieser Form der Kalzifikation kommt es zu Ein- oder Auflagerungen eines Kalziumphosphats. Eine sekundäre IOL-Kalzifikation kann grundsätzlich in jeder hydrophilen IOL unabhängig von ihrem Hersteller auftreten. Es wird vermutet, dass v. a. eine Veränderung der Zusammensetzung des Kammerwassers für die Entstehung verantwortlich ist. Nakanome et al. postulierten 2007, dass in Augen mit Konzentrationsschwankungen von Kalzium, Phosphat und Albumin im Kammerwasser infolge eines Zusammenbruchs der Blut-Kammerwasser-Schranke die Kalzifikation hydrophiler IOL begünstigt wird [23]. Andere Autoren sehen eher die Beschaffenheit des Kammerwasserflusses als entscheidend für die Entstehung der Pathologie an [1]. Oftmals sind Begleiterkrankungen oder im Anschluss an die Linsenoperation durchgeführte operative Eingriffe (z. B. posteriore lamelläre Keratoplastik, Vitrektomie, intravitreale Injektionen, rtPA-Eingabe in die Vorderkammer) für eine sekundäre Kalzifikation verantwortlich [11, 21, 27, 35]. So analysierten Schrittenlocher et al. retrospektiv 564 Patienten, bei denen nach gleichzeitiger oder vorheriger IOL-Implantation eine Descemet-Membran-endotheliale Keratoplastik (DMEK) durchgeführt wurde. Sie fanden heraus, dass es in 2,5 % dieser Fälle zu einer IOL-Kalzifikation kam. Weiterhin bestand eine Assoziation zwischen dem Auftreten einer IOL-Kalzifikation und der Anzahl der erfolgten Re-Bubblings [29]. Zu ähnlichen Ergebnissen kamen Ahad et al. 2014 in einer Studie mit 154 Augen, bei denen eine DSAEK durchgeführt wurde und es in 9,7 % der Fälle zu einer Vorderflächenkalzifikation kam. Auch hier zeigte sich eine deutlich höhere Kalzifikationsrate in Augen, bei denen ein Re-Bubbling erfolgte [2]. Makroskopisch konzentrieren sich die Eintrübungen der IOL bei dieser Form der Kalzifikation in der Regel nur auf den zentralen Bereich der Optik, also genau den Bereich, der von der Kapsulorhexis ausgespart ist (Abb. 3). Möglicherweise wird die Linsenoberfläche durch den Kontakt mit dem veränderten Kammerwasser und/oder der auslösenden Noxe (z. B. Gas) in dem Bereich derart verändert, dass die Entstehung von Kristallisationskeimen begünstigt wird [22]. Der genaue Pathomechanismus der sekundären Kalzifikation ist jedoch noch nicht verstanden. Eine kürzlich veröffentlichte Arbeit untersuchte Fälle, in denen 2 hydrophile IOL (1 Kapselsack-IOL und 1 additive IOL im Sulcus ciliaris) in ein Auge implantiert wurden und es zur Kalzifikation bei mindestens einer der Linsen kam. Interessanterweise trübten in einigen Fällen nur die Kapselsacklinse, in anderen Fällen nur die additive IOL und in wieder anderen Fällen beide Linsen ein. Neben Komorbiditäten und Folgeoperation im Anschluss an die Linsenimplantationen scheinen das Auftreten und der Schweregrad der Pathologie stark mit dem lokalen Umgebungsmilieu der Kunstlinse zusammenzuhängen [38]. Licht- und rasterelektronenmikroskopisch können auch bei sekundär kalzifizierten IOL unterhalb oder auf der Oberfläche zahlreiche feine, granuläre, kristallähnliche Ablagerungen aus Kalziumphosphat nachgewiesen werden, die in einer zu der Oberfläche parallelen Linie angeordnet sind [13]. Typischerweise ist aber – im Gegensatz zur primären Kalzifikation – nur eine der Oberflächen, meistens die Vorderseite der IOL, betroffen. Mittels energiedispersiver Röntgenspektroskopie kann gezeigt werden, dass es sich auch hier bei dem eingelagerten Material um ein Kalziumphosphat handelt (Abb. 4).

### Pseudokalzifikation

Die falsch positive Form bezieht sich auf solche Fälle, in denen andere Pathologien mit einer Kalzifikation verwechselt werden [12]. Besonders ein flüssiger Nachstar kann leicht mit einer primären Kalzifikation verwechselt werden. Auf eine gründliche Unterscheidung ist jedoch insbesondere im Hinblick auf die Frage einer Nd:YAG-Kapsulotomie (YAG-KT) zu achten. Während bei einem flüssigen Nachstar die YAG-KT die Trübung beseitigen kann, ist im Falle einer IOL-Kalzifikation von einer solchen abzusehen, da dadurch der operative Austausch der getrübten IOL erschwert wird [12]. Auch falsch positive Ergebnisse bei histologischen Färbungen werden als Pseudokalzifikation bezeichnet.

## Glistenings und Nanoglistenings in IOL aus hydrophobem Acrylat

**Figure 5:**
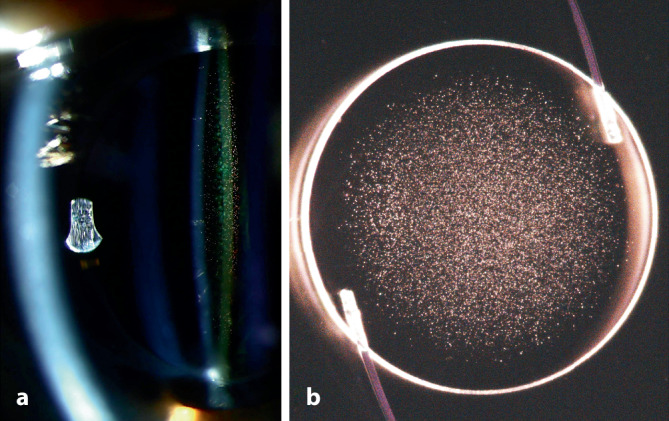

Glistenings sind kleine, mit Flüssigkeit gefüllte Vakuolen, die sich im IOL-Material entwickeln können. Seit der Einführung von IOL aus faltbarem hydrophobem Acrylat wird diese Pathologie beobachtet [6, 8]. Vakuolen mit Durchmessern von weniger als 200 nm, die bis zu 120 μm unter der Oberfläche der IOL liegen, werden als Nanoglistening („subsurface nanoglistenings“ [SSNG]) bezeichnet. Die Gründe für das Auftreten dieser Pathologie wurden lange untersucht, und es werden verschiedene IOL- und patientenabhängige Faktoren diskutiert [8, 34]. Es ist bekannt, dass der niedrige Wassergehalt hydrophober Materialien die Entstehung von Glistenings begünstigen kann. Einige Linsenhersteller haben deshalb die Gleichgewichtsfeuchte ihrer Linsenmaterialien durch Änderungen an der Polymerzusammensetzung angehoben. So wurde in einem hydrophoben Acrylat (AcrySof, Alcon, Fort Worth, USA) das Phenylethylmethacrylat durch Hydroxyethylmethacrylat ersetzt, sodass die Gleichgewichtsfeuchte von unter 0,5 % auf 1,5 % angehoben wurde. In einer kürzlich veröffentlichten klinischen Studie untersuchten Oshika et al. 110 Augen nach Implantation dieses neueren Linsenmaterials. In den bis zu 9 Jahren Nachbeobachtungszeit wurden in keinem dieser Fälle Glistenings oder Nanoglistenings beobachtet [26]. Für eine abschließende Bewertung müssen natürlich weitere Studienergebnisse abgewartet werden. Da die Entwicklung von Glistenings klinisch einige Monate bis Jahre dauern kann, wurden verschiedene In-vitro-Modelle entwickelt, um diese Pathologie im Labor nachzubilden und zu untersuchen. Dabei wurde v. a. ein Modell verwendet, welches Glistenings durch Temperaturunterschiede induziert [37]. Die Anordnung und Zahl der Glistenings ist bei verschiedenen IOL-Materialien unterschiedlich (Abb. 5). Durch eine kontinuierliche Verbesserung im Herstellungsprozess konnte die Dichte von Glistenings bei neueren IOL deutlich reduziert werden. Einige IOL-Modelle werden sogar als „glisteningfrei“ bezeichnet.

## Einfluss auf die optische Abbildungsqualität

**Figure 6:**
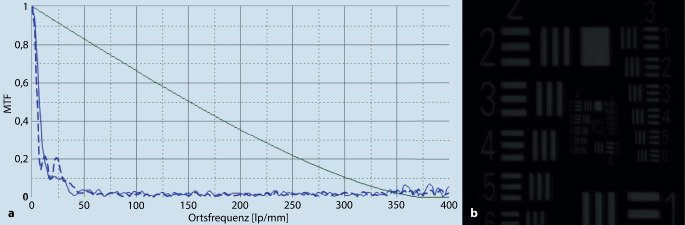

Die Kalzifikation von Kunstlinsen führt zu einer signifikanten Abnahme der Lichtdurchlässigkeit, einem Kontrastverlust und einer deutlichen Zunahme des Streulichtes, was die optische Qualität der IOL signifikant verschlechtern kann (Abb. 6).[19, 39] Laboruntersuchungen u. a. an der optischen Bank haben klären können, dass dabei der Visus nicht immer deutlich reduziert sein muss, durch das erhöhte Streulicht jedoch dennoch eine deutliche Beeinträchtigung der Sehqualität verursacht werden kann [39]. Es ist nach wie vor nicht vollständig geklärt, welchen Einfluss Glistenings auf die Sehfunktion haben, aber auch bei dieser Pathologie liegt das Ausmaß der Beeinträchtigung eher in einer Induktion von Streulicht [33]. Über die Notwendigkeit einer IOL-Explantation aufgrund von Glistenings wird allerdings nur sehr selten berichtet. Möglicherweise neigen Ophthalmologen jedoch dazu, die Bedeutung der Glistenings bei der Spaltlampenuntersuchung des vorderen Augenabschnittes zu unterschätzen; dies könnte daran liegen, dass die vom Patienten wahrgenommene Vorwärtsstreuung des Lichtes mehr als 300-mal stärker ist als die beobachtete Rückwärtsstreuung. Klinische Studien zeigen, dass Glistenings kaum die Sehschärfe beeinträchtigen, aber v. a. beim Nachtfahren mit Gegenlicht oder an einem sonnigen Tag Blendsymptome hervorrufen können. Der Einfluss von Glistenings auf die Sehqualität wurde auch mithilfe von In-vitro-Modellen untersucht. So konnte bei Untersuchungen an der optischen Bank gezeigt werden, dass eine recht hohe Anzahl von Glistenings erforderlich ist, um die optische Qualität (Modulationsübertragungsfunktion und Strehl-Ratio) zu verringern [33]. Glistenings scheinen allerdings Lichtstreuung zu verursachen und könnten so die Sehqualität durchaus beeinträchtigen. Dabei nimmt das Streulicht proportional zur Anzahl der Mikrovakuolen innerhalb der IOL-Optik zu [18].

## Therapie der Materialeintrübungen von Intraokularlinsen

In schweren Fällen einer Materialpathologie ist ein Austausch der IOL unausweichlich. Während kalzifizierte IOL wegen der deutlichen Beeinträchtigung der Sehqualität vergleichsweise häufig explantiert werden, wird über die Notwendigkeit einer IOL-Explantation aufgrund von Glistenings hingegen eher selten berichtet [25]. Bei IOL-Explantationen ist das Risiko eines Auftretens von Komplikationen (z. B. zonuläre Dehiszenz, Ruptur der hinteren Linsenkapsel, Dekompensation der Hornhaut) leider nicht immer zu vermeiden. In einer Fallserie mit 25 Augen eingetrübter Aqua-Sense-IOL berichteten Dagres et al. beispielsweise über das Auftreten von Komplikationen in 48 % der Fälle [5]. Insbesondere eine ausgeprägte Anhaftung der Haptiken und der Optik an der Linsenkapsel kann die Operation erschweren. Ein IOL-Austausch sollte daher nur bei deutlicher Symptomatik und nach besonders sorgfältiger Aufklärung des Patienten bezüglich des hohen Risikos erfolgen. Bei Patienten mit eingetrübten Kunstlinsen wird oftmals eine Nd:YAG-Laser-Kapsulotomie durchgeführt, da die Trübung als Nachstar fehlinterpretiert wird. Hiervon sollte aber dringend abgesehen werden, da eine eröffnete Hinterkapsel das Komplikationsrisiko bei einem IOL-Tausch unnötig erhöht. Leysen et al. präsentierten 2009 die chirurgischen Ergebnisse von 113 Fällen mit IOL-Austausch. Es zeigte sich, dass ein Glaskörpervorfall, der eine anteriore Vitrektomie erforderlich machte, stark mit einer präoperativen Nd:YAG-Laserkapsulotomie korrelierte. Eine anteriore Vitrektomie war in 18 von 37 Fällen mit einer präoperativen Nd:YAG-Laserkapsulotomie erforderlich, aber nur in 9 von 91 Fällen ohne präoperative Nd:YAG-Kapsulotomie [20]. Auch Versuche, die Trübungen mit einer „Laserpolitur“ abzutragen, sind bei Kalzifikationen von Linsen nicht Erfolg versprechend, da die Trübungen im Material der Intraokularlinsen liegen (Abb. 2 und 3). Nach Entfernung der eingetrübten IOL kann je nach Zustand des Kapselapparates eine neue IOL wieder im Kapselsack, im Sulcus ciliaris (idealerweise mit Durchführung eines „optic captures“, d. h. einem „Einknüpfen“ der Optik in die Rhexis), an der Iris oder an der Sklera fixiert werden.

## Rechtliche Aspekte

Jeder Fall einer IOL-Explantation muss dem Bundesinstitut für Arzneimittel und Medizinprodukte (BfArM) gemeldet werden. Linsenhersteller und -vertreiber bieten oft an, dass die explantierte IOL zur Untersuchung an die Firma zurückgesendet werden kann. Auch wenn eine gute Intention unterstellt werden sollte, so beinhaltet dieses Vorgehen einen grundsätzlichen Interessenkonflikt, da sich aus den Ergebnissen der Auswertung potenzielle Haftungsverpflichtungen ergeben könnten. Dies könnte die Aufarbeitung potenziell beeinträchtigen. Deshalb und auch im Sinne einer weiteren wissenschaftlichen Auswertung begrüßen wir die Einsendung des Explantats zusammen mit einem kurzen Formblatt an das unabhängige David J Apple Laboratory for Ocular Pathology (https://djapplelab.com/explant-iol-forms.html). Neben der BfArM-Meldung erfolgt dann zusätzlich eine strukturierte Erfassung und Aufarbeitung der Fälle. Wir möchten aber betonen, dass Linsenherstellern keinesfalls prinzipiell der Unwillen zur Aufarbeitung unterstellt werden sollte. Viele unserer Arbeiten wurden sogar v. a. an Explantaten durchgeführt, die uns die Hersteller und nicht die Chirurgen spezifisch zur weiteren Untersuchung zugeschickt haben [38].

Hersteller möchten sich z. T. durch Nennung bestimmter Themen im Beipackzettel gegen potenzielle Probleme absichern. So weisen einige Hersteller konkret darauf hin, dass die Eingabe von Luft oder Gas bei einem Hornhauteingriff oder einer Vitrektomie zur Kalzifikation der IOL führen kann. Der richtige Weg sollte jedoch nicht sein, die Verantwortung für das Problem auf den Arzt zu übertragen. In Deutschland besteht derzeit für Hersteller von Medizinprodukten keine gesetzliche Versicherungspflicht. Der Abschluss einer Haftpflichtversicherung ist hierzulande weiterhin freiwillig. Anders ist dies in anderen Ländern der Europäischen Union, wie beispielsweise in Frankreich. Dort besteht eine Versicherungspflicht für Medizinproduktehersteller (vgl. hierzu OLG Köln, Hinweisbeschluss vom 15.11.2017–5 U 68/17 [NJW-RR 2018, 868] „Beschränkung des Versicherungsschutzes einer Haftpflichtversicherung – Brustimplantate“). Obwohl auf europäischer Ebene bereits seit Längerem eine Vereinheitlichung des Medizinprodukterechts angestrebt wird, ist der Pflichtversicherungsschutz für Medizinprodukte bislang unionsrechtlich nicht harmonisiert worden (OLG Köln, Hinweisbeschluss vom 15.11.2017, a. a. O.). Auch das im Mai 2020 verkündete deutsche Medizinprodukte-EU-Anpassungsgesetz (MPEUAnpG) sieht eine solche Pflichtversicherung nicht vor. Zwar wurde teilweise im Gesetzgebungsverfahren die Einführung einer obligatorischen Produkthaftpflichtversicherung für die Hersteller gefordert. Diese Forderung hatte sich jedoch schlussendlich nicht durchgesetzt. Laut Stellungnahme des Gesamtverbands der Deutschen Versicherungswirtschaft spricht gegen die Einführung der Versicherungspflicht, dass die Versicherungsdichte auf freiwilliger Basis hierzulande besonders hoch ist. Die freiwillige Basis habe den Vorteil, dass der Versicherungsschutz individuell auf das jeweilige Haftungsrisiko zugeschnitten werden kann. Ferner ist laut Stellungnahme davon auszugehen, dass in Deutschland die Hersteller von Medizinprodukten seit dem Inkrafttreten des Artikel 10 Abs. 16 MDR und Artikel 19 Abs. 15 IVDR in aller Regel eine Haftpflichtversicherung abgeschlossen haben. Das Haftungsrisiko ist nämlich in der Regel im Rahmen einer Betriebshaftpflichtversicherung gedeckt, welche in Deutschland zum Versicherungsstandard gehört. Die Marktdurchdringung der Betriebshaftpflichtversicherung bei den deutschen Medizinproduktherstellern dürfte laut Stellungnahme bei nahezu 100 % liegen. Im Übrigen verbleibt es gemäß § 26 MPEUAnpG bei der Pflicht zum Versicherungsschutz zugunsten der von einer klinischen Prüfung oder einer sonstigen klinischen Prüfung betroffenen Person (Probandenversicherung), die dann für Schäden haftet, wenn bei der Durchführung der klinischen Prüfung ein Mensch getötet oder der Körper oder die Gesundheit eines Menschen verletzt wird.

### Infobox Mehr Informationen zum Thema

Auch der Vorstand der Deutschsprachigen Gesellschaft für Intraokularlinsen-Implantation, interventionelle und refraktive Chirurgie (DGII) empfiehlt, dass alle wegen Trübungen oder Diskolorierung explantierten IOL zur weiteren Untersuchung an das David J Apple Laboratory for Ocular Pathology (https://djapplelab.com/explant-iol-forms.html) gesandt werden sollten [7].

## Fazit für die Praxis

- Die heute auftretenden Materialeintrübungen von Intraokularlinsen (IOL) sind bei hydrophilen IOL v. a. die Kalzifikation und bei hydrophoben IOL die Glistenings.
- Die Art und das Ausmaß der Pathologie sind abhängig vom Linsenmaterial, von dem Herstellungsprozess der Linse sowie den lokalen Umgebungsbedingungen.
- In der Praxis sollte besonders auf die Lokalisation der Trübung geachtet werden (vor, innerhalb oder hinter der IOL), um Differenzialdiagnosen auszuschließen und die Patienten einer angemessenen Therapie zuzuführen.
- Die Unterscheide hinsichtlich der funktionellen Beeinträchtigung, die durch die verschiedenen Materialeintrübungen hervorgerufen werden, sollten bei der jeweiligen Therapieentscheidung berücksichtigt werden.
- Im Falle von beeinträchtigenden Materialeintrübungen besteht aktuell als einzige kausale Therapieoption der Austausch der IOL.
